# Supplementary material for: Application of a Fusion Method for Gas and Particle Air Pollutants between Observational Data and Chemical Transport Model Simulations Over the Contiguous United States for 2005–2014
Source: Int J Environ Res Public Health. 2019 Sep 9;16(18):3314. doi: 10.3390/ijerph16183314 (PMC6765984; doi:10.3390/ijerph16183314)
Supplement: Supplementary file 1 [file ijerph-16-03314-s001.pdf]

Supplementary Information

The following are available online at www.mdpi.com/xxx/s1.

Section S1. Metadata for 12km CMAQv5.0.2. Simulations

https://www.epa.gov/*CMAQ*/*CMAQ*-metadata)

Table S1. Regression values calculated for each year in the 2005–2014 domain for the 12 species. α represents the coefficient term, while β represents the exponential term. R^2 represents goodness of fit for the regression.

	α	β	R^2	α	β	R^2	α	β	R^2
	EC (ug/m^3)			OC (ug/m^3)			SO_4^{2-} (ug/m^3)		
**2005**	0.7	0.5	0.43	1.6	0.4	0.29	1.3	0.9	0.90
**2006**	0.7	0.4	0.43	1.5	0.4	0.21	1.1	1.0	0.90
**2007**	0.8	0.3	0.26	1.5	0.4	0.23	1.3	1.0	0.93
**2008**	0.8	0.4	0.28	1.5	0.5	0.35	1.2	0.9	0.83
**2009**	0.6	0.5	0.49	1.7	0.3	0.29	1.3	0.8	0.86
**2010**	0.6	0.4	0.41	1.7	0.3	0.23	1.1	0.9	0.82
**2011**	0.6	0.4	0.43	1.8	0.2	0.11	1.2	1.0	0.87
**2012**	0.6	0.5	0.37	1.8	0.2	0.10	1.1	0.9	0.85
**2013**	0.7	0.5	0.43	1.6	0.3	0.26	1.2	0.8	0.80
**2014**	0.6	0.5	0.33	1.6	0.3	0.20	1.2	1.0	0.77
	NO_3^- (ug/m^3)			NH_4^+ (ug/m^3)			$PM_{2.5}$ (ug/m^3)		
**2005**	1.3	0.5	0.43	1.3	0.6	0.7	4.2	0.4	0.50
**2006**	1.1	0.7	0.59	1.2	0.7	0.8	4.2	0.4	0.39
**2007**	1.2	0.8	0.60	1.5	0.7	0.7	4.0	0.4	0.39
**2008**	1.1	0.6	0.50	1.2	0.6	0.7	4.6	0.3	0.36
**2009**	1.1	0.7	0.50	1.2	0.5	0.6	4.4	0.3	0.33
**2010**	0.9	0.9	0.57	1.0	0.7	0.7	4.4	0.3	0.30
**2011**	0.9	0.9	0.54	1.0	0.6	0.5	4.2	0.4	0.33
**2012**	0.9	0.8	0.52	0.7	0.6	0.5	4.2	0.3	0.32
**2013**	1.0	0.8	0.46	0.7	0.5	0.2	4.4	0.3	0.28
**2014**	1.1	0.8	0.54	1.0	0.8	0.6	3.7	0.4	0.37
	PM_{10} (ug/m^3)			NO_2 (ppb)			NO_X (ppb)		
**2005**	12.1	0.2	0.1	2.2	0.7	0.63	4.2	0.6	0.48
**2006**	13.6	0.2	0.1	2.0	0.7	0.69	3.0	0.7	0.59
**2007**	14.1	0.2	0.1	1.8	0.8	0.66	2.7	0.7	0.58
**2008**	15.6	0.1	0.0	2.0	0.7	0.64	3.2	0.7	0.57
**2009**	13.9	0.1	0.0	1.8	0.7	0.69	2.4	0.7	0.60
**2010**	14.5	0.1	0.0	1.7	0.8	0.69	2.2	0.7	0.63

**2011**	12.0	0.2	0.1	2.2	0.7	0.68	2.5	0.8	0.64
**2012**	14.6	0.1	0.0	2.2	0.7	0.69	2.6	0.7	0.63
**2013**	11.7	0.2	0.1	2.0	0.7	0.70	2.0	0.9	0.65
**2014**	11.7	0.2	0.1	2.1	0.7	0.71	2.4	0.8	0.64
	**SO_2 (ppb)**			**O_3 (ppm)**			**CO (ppm)**		
**2005**	2.2	0.7	0.38	0.4	0.7	0.59	1.1	0.4	0.20
**2006**	1.9	0.9	0.53	0.4	0.7	0.58	1.1	0.4	0.18
**2007**	1.5	0.9	0.54	0.5	0.8	0.65	1.0	0.5	0.22
**2008**	3.6	0.7	0.42	0.5	0.8	0.59	0.9	0.5	0.24
**2009**	1.5	0.9	0.47	0.4	0.7	0.51	0.9	0.5	0.21
**2010**	1.4	0.9	0.44	0.4	0.7	0.52	0.9	0.5	0.16
**2011**	1.7	0.8	0.42	0.3	0.6	0.53	0.8	0.5	0.20
**2012**	1.5	0.8	0.43	0.7	0.9	0.58	0.8	0.5	0.24
**2013**	1.5	0.7	0.37	0.5	0.8	0.50	1.0	0.7	0.33
**2014**	1.5	0.9	0.46	0.5	0.8	0.54	1.1	0.6	0.36

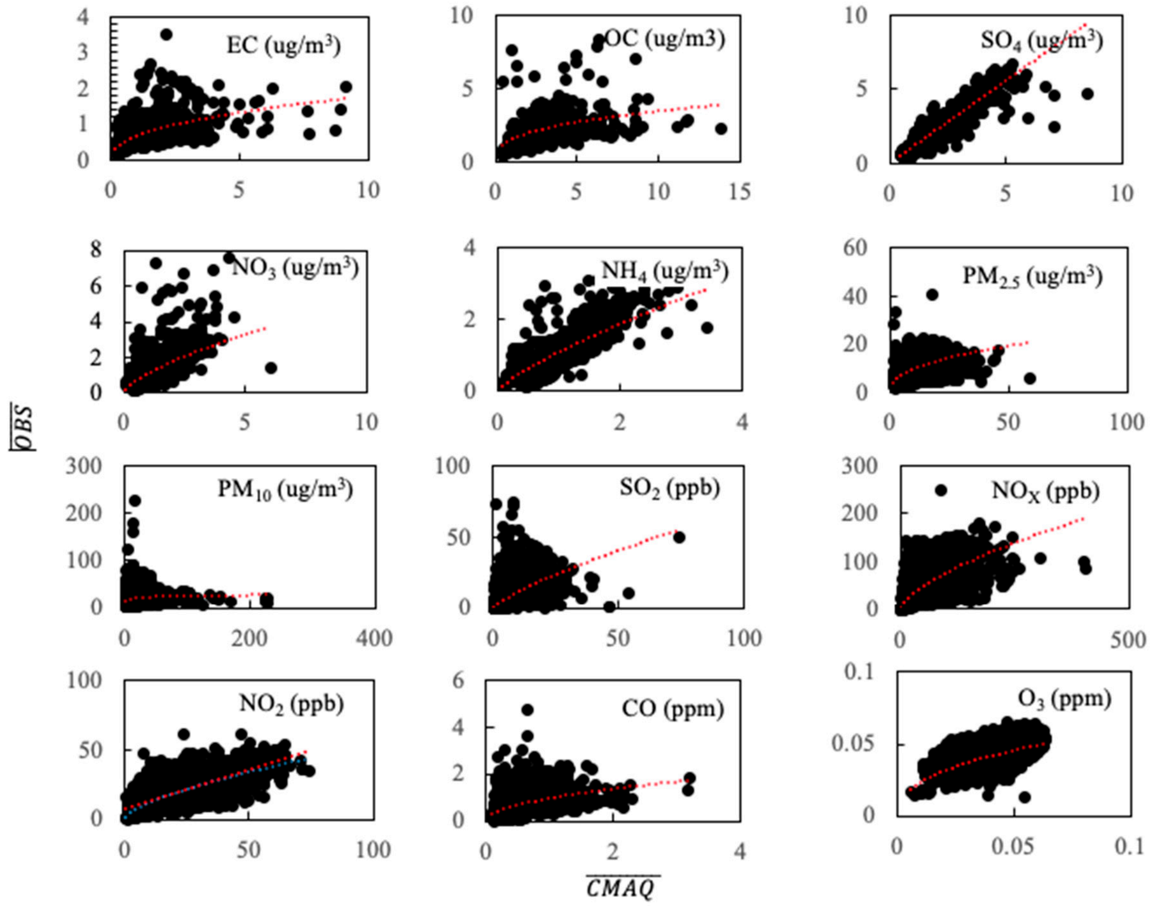

Figure S1. Regression plots for the data between 2005–2014. The x-axis represents the annual averaged *CMAQ*, and the y-axis represents the annual averaged observations. The *CMAQ* and observations are averaged according to Equations (2a) and (2b).

Table S2. A comparison of the R^2 and NRMSE for the spatial and temporal performance. The fused columns are the metrics for the fused field, and the *CMAQ* columns are the metrics for the *CMAQ*

output. The columns titled temporal show the temporal metrics, and the column titled spatial show the spatial metrics. The metrics shown are averaged over the ten-year time span.

	**Temporal**				**Spatial**			
	**R^2**		**NRMSE**		**R^2**		**NRMSE**	
	**Fused**	**CMAQ**	**Fused**	**CMAQ**	**Fused**	**CMAQ**	**Fused**	**CMAQ**
**$PM_{2.5}$**	0.96	0.38	0.07	0.43	0.77	0.32	0.25	0.46
**EC**	0.99	0.41	0.03	0.50	0.74	0.39	0.33	0.52
**OC**	0.99	0.32	0.02	0.48	0.77	0.29	0.28	0.51
**NO_3^-**	0.99	0.49	0.05	0.76	0.80	0.62	0.37	0.56
**NH_4^+**	0.99	0.47	0.03	0.58	0.83	0.49	0.32	0.57
**SO_4^{2-}**	0.99	0.49	0.03	0.46	0.92	0.73	0.10	0.21
**PM_{10}**	0.90	0.21	0.15	0.50	0.44	0.05	0.67	0.88
**CO**	0.91	0.21	0.14	0.54	0.46	0.11	0.56	0.73
**NO_2**	0.95	0.26	0.07	0.39	0.74	0.44	0.33	0.48
**NO_X**	0.96	0.25	0.12	0.68	0.61	0.26	0.61	0.86
**SO_2**	0.84	0.15	0.39	1.07	0.42	0.09	1.44	1.80
**O_3**	0.99	0.51	0.02	0.20	0.84	0.41	0.10	0.19

Table S3. A comparison of spatiotemporal R^2 and NRMSE calculated for the 10% data withholding evaluation of the fused fields. The metrics shown are the averaged results over the ten-year time span.

	**Evaluation**	
	**R^2**	**NRMSE**
**$PM_{2.5}$**	0.74	0.26
**EC**	0.55	0.43
**OC**	0.59	0.38
**NO_3^-**	0.64	0.60
**NH_4^+**	0.69	0.43
**SO_4^{2-}**	0.68	0.35
**PM_{10}**	0.57	0.37
**CO**	0.42	0.44
**NO_2**	0.46	0.02
**NO_X**	0.46	0.52
**SO_2**	0.21	1.04
**O_3**	0.83	0.11

Table S4. Linear regression results for the population weighted concentrations. The units of the slope are dimensionless, and the units of the intercept correspond to the units of the pollutant presented in Figure 5.

	**Slope**	**Intercept**
**$PM_{2.5}$**	-1.1E-03	11.72
**EC**	-1.0E-04	0.79

**OC**	-2.0E-05	2.00
**NO_3^-**	-6.0E-05	1.45
**NH_4^+**	-3.0E-04	1.44
**SO_4^{2-}**	-6.0E-04	3.36
**PM_{10}**	-1.3E-03	22.47
**CO**	-1.0E-04	0.77
**NO_2**	-1.8E-03	21.44
**NO_X**	-4.8E-03	44.36
**SO_2**	-1.8E-03	77.82
**O_3**	-6.0E-07	0.04
